# Betulinic Acid Attenuates Osteoarthritis via Limiting NLRP3 Inflammasome Activation to Decrease Interleukin-1*β* Maturation and Secretion

**DOI:** 10.1155/2023/3706421

**Published:** 2023-09-25

**Authors:** Bo Liu, Yanglin Wu, Ting Liang, Yunlong Zhou, Guangdong Chen, Jiaheng He, Chenchen Ji, Peixin Liu, Chenhui Zhang, Jun Lin, Kece Shi, Zongping Luo, Naicheng Liu, Xinlin Su

**Affiliations:** ^1^Department of Orthopaedics, People's Hospital of Leshan, 238 Baita Road, Leshan 614000, Sichuan, China; ^2^Department of Orthopaedics, First Affiliated Hospital of Soochow University, 899 Pinghai Road, Suzhou 215006, Jiangsu, China; ^3^Orthopaedic Institute, Soochow University, 708 Renmin Road, Suzhou 215006, Jiangsu, China; ^4^Department of Orthopaedics, Tenth People's Hospital of Tongji University, 301 Middle Yanchang Road, Shanghai 200072, Shanghai, China; ^5^Department of Orthopaedics, Jiangsu Shengze Hospital, No. 1399, Market West Road, Shengze 215000, Jiangsu, China; ^6^Stroke Intensive Care Unit, Children's Hospital of Soochow University, 92 Zhongnan Road, Suzhou 215006, Jiangsu, China; ^7^Department of Orthopedics, Suzhou Xiangcheng People's Hospital, 1060 Huayuan Road, Suzhou 215131, Jiangsu, China; ^8^Department of Orthopaedics, Suzhou Dushu Lake Hospital, Dushu Lake Hospital Affiliated to Soochow University, Medical Center of Soochow University, Suzhou 215001, Jiangsu, China

## Abstract

**Introduction:**

Osteoarthritis (OA) is the most common degenerative joint disorder. Prior studies revealed that activation of NLRP3 inflammasome could promote the activation and secretion of interleukin-1*β* (IL-1*β*), which has an adverse effect on the progression of OA. Betulinic acid (BA) is a compound extract of birch, whether it can protect against OA and the mechanisms involved are still unknown.

**Materials and Methods:**

In vivo experiments, using gait analysis, ELISA, micro-CT, and scanning electron microscopy (SEM), histological staining, immunohistological (IHC) and immunofluorescence (IF) staining, and atomic force microscopy (AFM) to assess OA progression after intraperitoneal injection of 5 and 15 mg/kg BA in an OA mouse model. In vitro experiments, caspase-1, IL-1*β*, and the N-terminal fragment of gasdermin D (GSDMD-NT) were measured in bone marrow-derived macrophages (BMDMs) by using ELISA, western blot, and immunofluorescence staining.

**Results:**

We demonstrated that OA progression can be postponed with intraperitoneal injection of 5 and 15 mg/kg BA in an OA mouse model. Specifically, BA postponed DMM-induced cartilage deterioration, alleviated subchondral bone sclerosis, and relieved synovial inflammation. In vitro studies, the activated NLRP3 inflammasome produces mature IL-1*β* by facilitating the cleavage of pro-IL-1*β*, and BA could inhibit the activation of NLRP3 inflammasome in BMDMs.

**Conclusions:**

Taken together, our analyses revealed that BA attenuates OA via limiting NLRP3 inflammasome activation to decrease the IL-1*β* maturation and secretion.

## 1. Introduction

Osteoarthritis (OA) is the most common degenerative joint disorder, affecting more than 240 million people worldwide [1–3]. In adults, OA is the foremost reason for restricted activity [1]. Currently, treatments for OA focus on joint function amelioration and pain symptom mitigation; however, these methods cannot halt disease progression or reverse any damage [3, 4]. Articular cartilage deterioration, subchondral bone reconstruction, and synovitis play a crucial role during the OA development [5, 6]. The degradation of cartilage is probably due to the lack of sufficient extracellular matrix (ECM) of cartilage, the major components of which are aggrecan (ACAN) and collagen II (COL II) [3]. COL II is the most important structural protein of cartilage and regulates the metabolic balance. ACAN is embedded in the molecular structure of cartilage, can absorb water into the cartilage, and has an antipressure effect [7, 8]. In OA, the internal environment of chondrocytes changes, water loss occurs in articular cartilage, and the expression of COL II and ACAN decreases, leading to structural changes in the cartilage. Moreover, the expression of collagen X (COL X) and matrix metalloproteinase-13 (MMP-13) increases, which further aggravates the degeneration of the articular cartilage [9, 10].

The NLRP3 inflammasome comprises NLRP3, ASC, and pro-caspase-1, of which, ASC (apoptosis-associated speck-like protein) contains a caspase recruitment domain [11]. Through activated caspase-1, the activated NLRP3 inflammasome produces mature IL-1*β* by facilitating the cleavage of pro-IL-1*β*. Caspase-1 is important for the cleavage of gasdermin D (GSDMD), which leads to the formation of the N-terminal fragment (GSDMD-NT) [11]. By pores in the plasma membrane, GSDMD-NT mediates the secretion of IL-1*β* [12]. The NLRP3 inflammasome levels and the progression of OA might be correlated [13]. Pro-inflammatory cytokines are critical mediators affecting the pathophysiological development of OA, especially IL-1*β*, which controls the degeneration of the articular cartilage matrix and can initiate and spread inflammation either alone or jointly with other cytokines [14]. The ability of IL-1*β* to downregulate the level of alpha 1(II) procollagen mRNA and suppress the synthesis of COL II in cultured human chondrocytes, and can lead to the inhibition of the synthesis of ACAN in rat chondrocytes [15, 16]. Additionally, IL-1*β* stimulates chondrocytes to release several enzymes, including MMPs and ADAMTS (a disintegrin-like and metalloproteinase with thrombospondin type 1 motifs). MMP-13 and ADAMTS-4, in particular, are crucial to cartilage damage mediation [17–19]. Therefore, there is a strong association between IL-1*β* and the progression of OA. Kobayashi et al. [20] showed that inhibition of IL-1*β* secretion has a beneficial effect on the progression relief in OA. Therefore, the inhibition of IL-1*β* production by the prevention of NLRP3 inflammasome activation is a potential treatment for OA.

Betulinic acid (BA) is a compound extract of birch, which has anti-inflammatory effects [21, 22]. Whether it can protect against OA and the mechanisms involved are still unknown. Therefore, investigating the effect of BA on OA is indispensable. In this study, we hypothesized that the OA-triggered cartilage deterioration could be mitigated by BA, by deactivating the NLRP3 inflammasome to suppress the production of IL-1*β*. We investigated whether BA could inhibit IL-1*β* production in vitro and determined its causes. In vivo, mice with OA, which were induced by the destabilization of the medial meniscus (DMM) surgery, were administered intraperitoneal BA to assess how it postponed the cartilage deterioration.

## 2. Materials and Methods

### 2.1. Experimental Animals and BA Treatment

Eighty-eight 8-week-old male C57BL/6J mice were procured from the JOINN Laboratories (Suzhou), Inc. (License No. SCXK (Su) 2018–0006, Suzhou, China). After acclimatization for 1 week, the mice were placed in a room at 23 ± 2°C and 55% ± 10% RH, with a 12-hr light (7:00 am–7:00 pm) and 12-hr dark schedule. The mice were provided *ad libitum* access to food and water.

**Figure 1:**
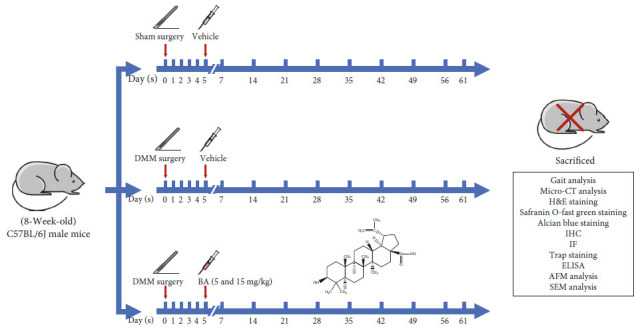
The molecular architecture of BA and the schematic of the timeline of the experiment.

The mice were randomly placed in one of four groups. Twenty mice were placed in each group, which included the Sham + Vehicle group (Sham mice treated with vehicle solution), the DMM + Vehicle group (DMM mice treated with vehicle solution), the DMM + Low-dose group (DMM mice treated with 5 mg/kg BA), and the DMM + High-dose group (DMM mice treated with 15 mg/kg BA). Their right knee joints underwent DMM surgery as described in the previous studies [23, 24], whereas the mice in the Sham + Vehicle group underwent Sham operations. BA was dissolved in the vehicle solution (30% ethyl alcohol–saline solution). Five days after surgery, the mice in the DMM + Low-dose and DMM + High-dose groups were intraperitoneally injected with 0.1 mL BA once every other day till Week 8, while the mice in the Sham + Vehicle and DMM + Vehicle groups were administered an equal volume of vehicle solution (Figure 1).

### 2.2. Gait Analysis

**Figure 2:**
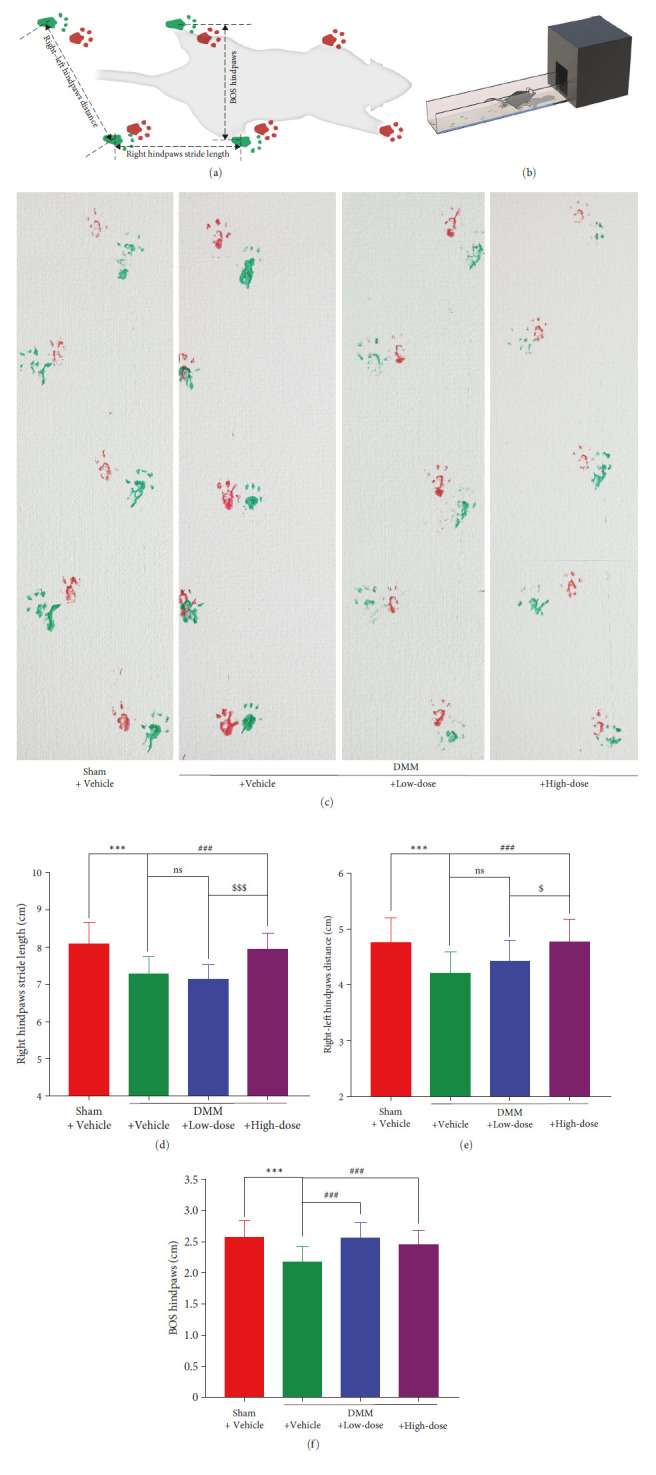
BA ameliorated gait in DMM-induced OA mice. (a) Graphical representation of selected gait parameters. (b) Schematic illustration of gait analysis. (c) Representative gait bands of mice. (d) Quantification of the right hindpaws stride length, (e) the right-left hindpaws distance, and (f) the BOS handpaws.  ^*∗∗*^*P* < 0.01 and  ^*∗∗∗*^*P* < 0.001 against Sham + Vehicle group; ^#^*P* < 0.05, ^##^*P* < 0.01, and ^###^*P* < 0.001 against DMM + Vehicle group; ^$^*P* < 0.05 and ^$$$^*P* < 0.001 against DMM + Low-dose group. (*n* = 16–20).

Gait recording was performed 8 weeks after treatment. Each mouse was placed on a walkway (10 cm wide and 50 cm long) and was allowed to walk back and forth freely. The forepaws of the mice were painted red, and the hind paws were painted green. A paper was used to record the footprints on the walkway. Finally, the base of support (BOS) hindpaws, the right hindpaws stride length, and the right–left hindpaws distance were quantified and analyzed and shown in Figures 2(a) and 2(b) [25].

### 2.3. Quantification of Serum IL-1*β* and Cartilage Oligomeric Matrix Protein (COMP)

The mice were anesthetized with 2% isoflurane. Following anesthesia, ocular blood was collected and allowed to clot for 1 hr at ambient temperature, and then centrifuged for 10 min at 4°C and 1,500 rpm. Next, serum was collected and stored at −80°C in aliquots. Finally, ELISA kits (IL-1*β*: MI038; COMP: MC040, Gefan, China) were used to quantify the IL-1*β* and COMP in the serum.

### 2.4. Micro-CT and SEM Analyses

After processing the murine right knee joints, a high-resolution SkyScan1176 micro-CT (Aartselaar, Belgium) was used for the sample analysis. The isometric resolution was set at 9 *µ*m, and the X-ray conditions included a voltage of 50 kV, an electric current of 200 *µ*A, and an Al filter of 0.5 mm. Subsequently, the NReon, Dataview, and CTan software were used to quantitatively analyze the bone mass-associated indices, as well as, the regions of interest (ROI), which was centered at the subchondral bone of the tibial medial plateau with 30 consecutive layers. Bone mass-related indicators, including the volumetric ratio of bone to tissue (BV/TV; %), bone mineral density (BMD; g/cm^3^), the thickness of the trabecula (Tb.Th; mm), as well as, the number of pores were recorded. Three-dimensional images were reconstructed using the Mimics software.

After processing, the right knee joints were digested using an enzymatic mixture (2% Type I collagenase mixed with 2% Type II collagenase) until complete digestion of the cartilage and peripheral soft tissues occurred. Then, the specimens were fixed with glutaraldehyde (4%) and dehydrated using an ethanol gradient. Finally, an FEI Quanta 250 scanning electron microscopy (SEM) (Hillsboro, OR, USA) was used to detect the microfractures in the ROI of the specimens.

### 2.5. Histological and Trap Staining Analyses

After analysis by micro-CT, the specimens were decalcified for 1 month in 10% ethylenediaminetetraacetic acid (EDTA) (pH 7.4) and then embedded in the paraffin. The right knee joints were cut into sections (6 *µ*m thick) and deparaffinized in the xylene. They were hydrated with graded ethanol and stained using kits such as safranin O-fast green, hematoxylin–eosin (H&E), alcian blue, and tartrate-resistant acid phosphatase (Trap) (Sigma–Aldrich, MO, USA). To determine the articular breakdown of the medial tibial plateau, the ratio of hyaline cartilage to calcified cartilage (HC/CC) and the Osteoarthritis Research Society International (OARSI) scoring protocol were followed [26–28]. A previously reported scoring protocol was followed for the assessment of synovitis [29]. Histomorphometric determinations of the articular cartilage and synovitis were performed based on the microphotographed sections. The number of Trap + osteoclasts (OCs) in per mm^2^ was quantified using the ImageJ software.

### 2.6. Immunohistological (IHC) and Immunofluorescence (IF) Staining Analyses

For immunohistological (IHC) staining, murine articular sections were deparaffinization and hydration, followed treated with parenzyme (0.25%) for 30 min. An additional 10-min treatment with H_2_O_2_ (3%) was also performed. Next, the sections were treated with goat serum (10%) for 20 min and incubated with primary antibodies at 4°C overnight. The secondary antibodies were selected based on the primary antibody host. Following visualization of the signals using the DAB kit, hematoxylin counterstaining was performed. During immunofluorescence (IF) staining, murine articular sections were treated at 97°C for 30 min using 1x sodium citrate, followed by deparaffinization and hydration, and a further 10-min treatment with H_2_O_2_ (3%) was performed. Then, the sections were treated with goat serum (10%) for 20 min and incubated overnight with primary antibodies at 4°C. After adding fluorescence-conjugated secondary antibodies; the sections were incubated with DAPI dye solution for 10 min at room temperature in the dark. The primary antibody of ACAN (Proteintech), MMP-13 (Proteintech), COL II (Santa), and COL X (Abcam). The sections were microphotographed using an Axiovert 40C optical microscope (Zeiss, Germany), and ImageJ software was used to quantify the percentage of the positive stained area in the total area.

### 2.7. AFM Analysis and Nanomechanical Testing

For cartilage, after euthanasia, the right tibial plateaus were isolated without ligament tissues and tendon, and then cyanoacrylate gel was used to glue them onto the atomic force microscopy (AFM) specimen discs. Throughout the measurement process, the samples were immersed in the PBS solution to minimize the cartilage degradation [30]. A borosilicate colloidal spherical tip (*R* = 5 *µ*m, *k* = 0.06 N/m) was used to perform AFM-based indentation (Dimension ICON, Bruker, MA, USA) on the cartilage surface of the tibial plateaus.

(1) $$\begin{array}{c} \begin{array}{c} E=\frac{\sqrt{\pi }}{2}\left(1-\upsilon^{2}\right)\frac{S}{\sqrt{A}} \end{array}. \end{array}$$

Here, *S* is the contact stiffness and *A* is the acreage.

For subchondral bone, the optimum cutting temperature (OCT) compound was used for embedding the right knee joint, and then, a CM^3^050S microtome (Leica, Nussloch, Germany) was used for sectioning the joints (20−30 *µ*m thick). AFM-based indentation was performed on the subchondral bone using a probe (*R* = 5 nm, *k* = 40 N/m). The compression modulus computation of the elastic modulus was performed based on the Hertz model.

(2) $$\begin{array}{c} F=\frac{4}{3}\frac{E}{\left(1-\upsilon^{2}\right)}\sqrt{R}\delta^{3/2}. \end{array}$$

Here, *F* denotes the pressure, *E* and *ν* refer to Young's modulus and Poisson's ratio, respectively, *R* denotes the tip radius, and *δ* is the indentation depth.

### 2.8. Cell Culture and IF Staining

Bone marrow-derived macrophages (BMDMs) were prepared as previously described [31–33]. BMDMs were primed with lipopolysaccharide (LPS) (500 ng/mL) (Sigma) for 4 hr and then stimulated with ATP (3 mM) (MedChemExpress) or ATP + BA for 1 hr. Then, the cells were used for the further study. The THP-1 cells were purchased from Procell (Wuhan, China).

The Calcein/PI staining were performed with a Calcein/PI Cell Viability kit according to the instructions of manufacturer.

### 2.9. Western Blot

After intervention, the supernanants and cell lysates were collected. Western blot was performed as previously described [31–33]. The primary antibody of ASC (Abcam), caspase-1 (Cell Signaling Technology), IL-1*β* (Abcam), and GSDMD (Abcam) was used. The result was quantified by ImageJ.

### 2.10. Statistical Analysis

Data were analyzed statistically using Prism 8.0 (GraphPad Software, CA, USA). All data are expressed as the mean ± SD. One-way analysis of variance (ANOVA) and Tukey's post hoc test were performed to determine the differences among the groups. The differences were considered to be statistically significant for *P* < 0.05.

## 3. Results

### 3.1. BA Ameliorated Gait in DMM-Induced OA Mice

To determine how BA potentially affects the activities in DMM mice, we collected the footprints for gait analysis 8 weeks after BA intervention (Figure 2(c)). After DMM operation, the right hindpaws stride length, the right–left hindpaws distance, and the BOS handpaws were measured, and the differences were significant (*P* < 0.001). Nonsignificant changes were noted in the right hindpaws stride length and the right–left hindpaws distance after low-dose BA intervention (*P* > 0.05), although a prominent increase in the mice of the DMM + High-dose group was observed compared to that in the mice of the DMM + Vehicle group (*P* < 0.001). The BOS handpaws increased after intervention with both low and high doses of BA (*P* < 0.001). Additionally, for both the right handpaws stride length and the right–left handpaws distance, the DMM + High-dose mice outperformed the DMM + Low-dose mice (Figure 2(d)–2(f)).

### 3.2. The Contents of IL-1*β* and COMP in the Serum of DMM Mice Decreased after BA Treatment

**Figure 3:**
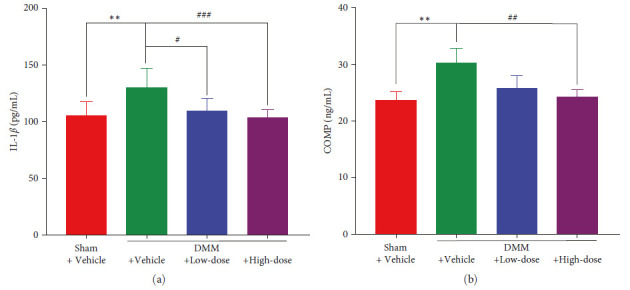
The contents of IL-1*β* and COMP in the serum of DMM mice decreased after BA treatment. The contents of IL-1*β* in the serum. (*n* = 6–12). (b) serum levels of the COMP. (*n* = 3–5).  ^*∗∗*^*P* < 0.01 against Sham +Vehicle group; ^#^*P* < 0.05, ^##^*P*<0.01, and ^###^*P* < 0.001 against DMM + Vehicle group.

After 8 weeks of the BA intervention, peripheral blood was collected and upper serum was extracted. Then, the ELISA kit was used for determining the serum levels of IL-1*β* and COMP. The mice in the DMM + Vehicle group exhibited significantly higher IL-1*β* and COMP (*P* < 0.01) than those in the mice of the Sham +Vehicle group. Compared to that in the DMM + Vehicle group, IL-1*β* decreased significantly in the DMM + Low-dose group (*P* < 0.05) and the DMM + High-dose group (*P* < 0.001). Although a nonsignificant change was found in COMP between the DMM + Low-dose and the DMM + Vehicle groups, COMP in the DMM + High-dose group was significantly lower than that in the DMM + Vehicle group (*P* < 0.01) (Figure 3).

### 3.3. BA Relieved the Synovial Inflammation Induced by DMM Surgery

**Figure 4:**
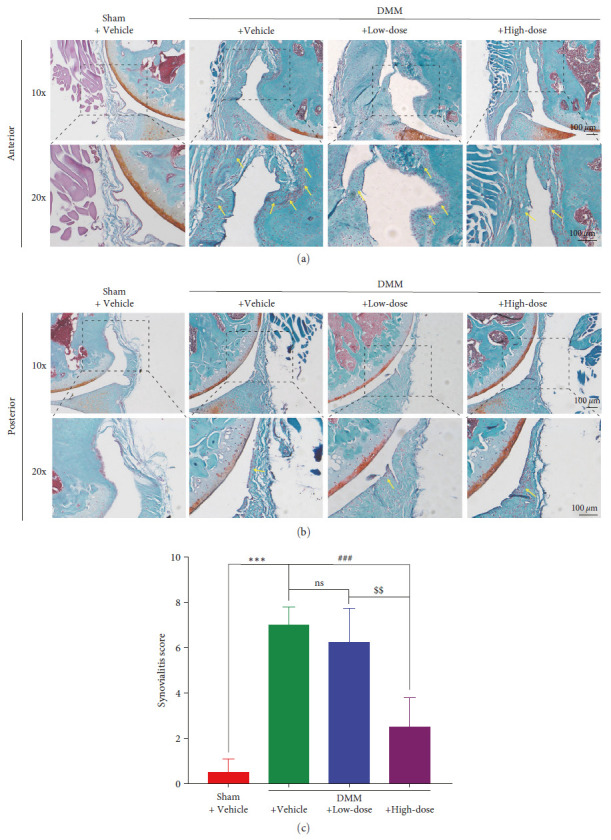
BA relieved the synovial inflammation induced by DMM surgery. (a) Safranin O-fast green staining of the murine anterior knee synovium and (b) the murine posterior knee synovium. (c) The synovialitis scores for synovial inflammation in mice. Yellow arrows indicate the infiltration of inflammatory cells.  ^*∗∗∗*^*P* < 0.001against Sham +Vehicle group; ^###^*P* < 0.001 against DMM + Vehicle group; ^$$^*P* < 0.01 against DMM + Low-dose group; ns: no significance; scale bar = 100 *µ*m. (*n* = 3).

To determine the attenuation of synovial inflammation after BA treatment, staining with Safranin O and Fast green was performed. Intra-articular synovial hyperplasia and abundant cell infiltration were observed in the DMM + Vehicle group. Although in the DMM + Low-dose group, intra-articular synovial hyperplasia and cell infiltration did not change significantly, synovial inflammation was effectively alleviated after treatment with a high dose of BA (Figures 4(a) and 4(b)). The results showed that the DMM + Vehicle mice scored significantly higher for the synovial inflammation than the Sham + Vehicle mice (*P* < 0.001). There was no significant reduction in the synovial inflammation scores of the DMM + Low-dose mice compared to those of the DMM + Vehicle mice (*P* > 0.05), while significant reductions in the scores were noted in the DMM + High-dose mice (*P* < 0.001) (Figure 4(c)).

### 3.4. BA Postponed DMM-Induced Articular Cartilage Deterioration in Mice

**Figure 5:**
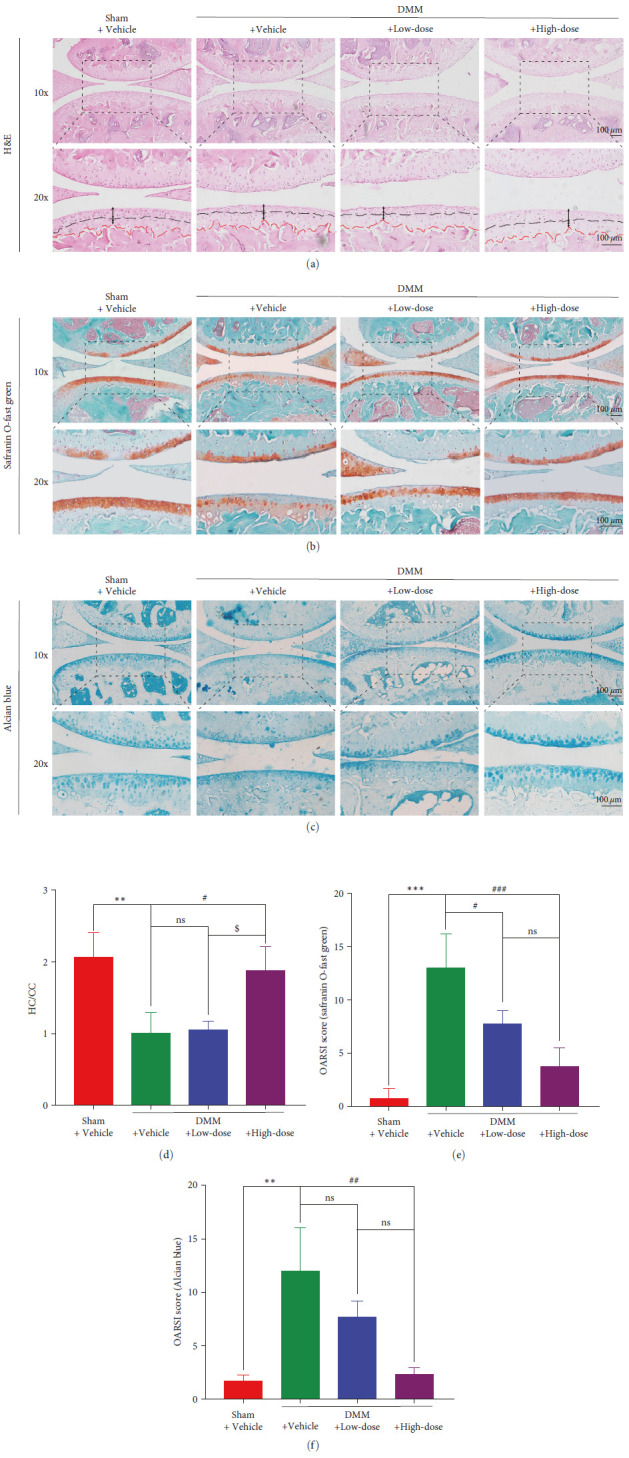
BA postponed DMM-induced articular cartilage deterioration in mice. (a) H&E staining (thickness of HC and CC were marked by double-headed arrows), (b) Sagittal safranin O-fast green staining, and (c) Alcian blue staining of the cartilage in the right knee. (d) Quantification of articular cartilage damage by H&E staining, evaluated from the HC/CC ratio. (*n* = 3). (e) OARSI score-based quantification of articular cartilage damage in safranin O-fast green staining. (*n* = 4). (f) Quantification of the sulfated GAG chains of proteoglycans in alcian blue staining, evaluated by the OARSI score. (*n* = 3).  ^*∗∗*^*P* < 0.01,  ^*∗∗∗*^*P* < 0.001 vs. Sham + Vehicle group; ^#^*P* < 0.05, ^##^*P* < 0.01, and ^###^*P* < 0.001 vs. DMM + Vehicle group; ^$^*P*<0.05 vs. DMM + Low-dose group; ns: no significance; scale bar = 100 *µ*m.

To determine the attenuation of cartilage degeneration after BA treatment, staining with H&E, alcian blue, and safranin O-fast green was performed (Figure 5(a)–5(c)). For H&E staining, the HC/CC ratio of the right knee joint in the mice was quantitatively analyzed. The thickness of HC in the DMM + Vehicle group was thinner, and the HC/CC ratio decreased significantly than that in the Sham + Vehicle group (*P* < 0.01). Nonsignificant differences were found between DMM + Low-dose and DMM + Vehicle mice for the HC/CC ratio and HC thickness (*P* > 0.05). The HC thickness in the DMM + High-dose group was significantly higher than that in the DMM + Vehicle group, and the HC/CC ratio was also significantly higher (*P* < 0.05) (Figure 5(d)). The cartilage histopathology of the mice was acquired by safranin O-fast green staining and quantitatively analyzed by the OARSI scoring system. The OARSI score of the DMM + Vehicle group was 13.000 ± 3.162, which was significantly higher than that of the Sham + Vehicle group (0.750 ± 0.957) (*P* < 0.001). The DMM + Low-dose mice (7.750 ± 1.258) exhibited a significantly lower OARSI score (*P* < 0.05) than the DMM + Vehicle mice. The DMM + High-dose mice had a lower OARSI score than the DMM + Vehicle mice (*P* < 0.001) (Figure 5(e)). Additionally, we stained the sulfated glycosaminoglycan (GAG) chains of proteoglycans with alcian blue and conducted a quantitative analysis. The results suggested that the DMM + Vehicle group had a significantly higher OARSI score (*P* < 0.01) than the Sham + Vehicle group. A nonsignificant change was found in the DMM + Low-dose group compared to the DMM + Vehicle group for the OARSI scores (*P* > 0.05), and the OARSI scores in the DMM + High-dose group was significantly lower compared to the DMM + Vehicle group (*P* < 0.01) (Figure 5(f)).

### 3.5. BA Attenuated DMM-Induced Degradation of Mice Knee Cartilage Matrix

**Figure 6:**
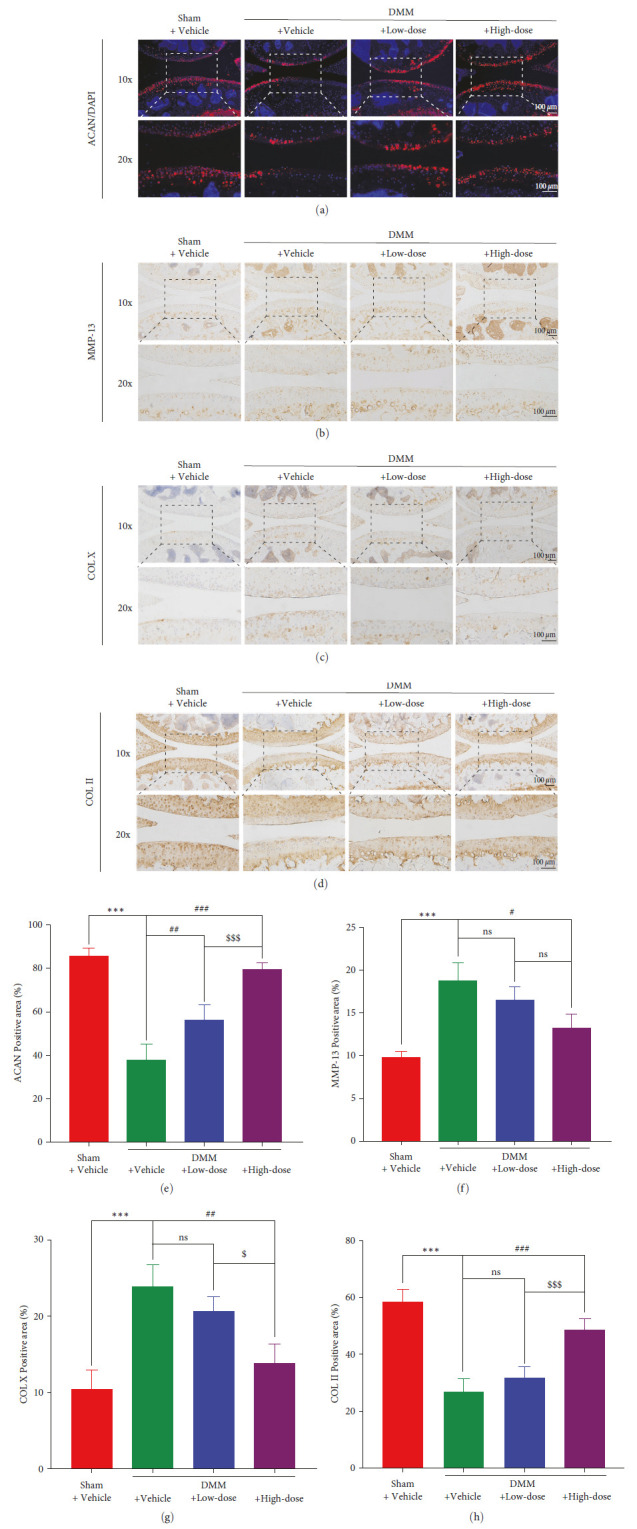
BA protected against DMM-induced degradation of mice knee cartilage matrix. (a) Sagittal pictures of cell nucleus (blue), ACAN (red), and mergence in the knee cartilage of mice. (b) IHC sagittal images of MMP-13 and IHC sagittal images of (c) COL X and (d) COL II. (e) Quantification of the positive area of ACAN expression (*n* = 3–5), and quantification of the positive area of (f) MMP-13 (*n* = 3), (g) COL X (*n* = 3–5), and (h) COL II expression (*n* = 4–6).  ^*∗∗∗*^*P* < 0.001 vs. Sham + Vehicle group; ^#^*P* < 0.05, ^##^*P* < 0.01, ^###^*P* < 0.001 vs. DMM + Vehicle group; ^$^*P* < 0.05, ^$$$^*P*<0.001vs. DMM + Low-dose group; ns: no significance; scale bar = 100 *µ*m.

The articular cartilage matrix is composed of several components, mainly including ACAN and COL II, which work together to maintain the functionality of the joint cartilage. In OA, the release of MMP-13 and COL X from the cartilage matrix increases [9]. To evaluate the severity of cartilage damage after DMM surgery and the protective effects of BA on cartilage, we performed IF and IHC staining on mice knee specimens. For IF staining, the joint cartilage levels of ACAN in mice decreased significantly following the DMM surgery, while the expression of ACAN increased after treatment with the different concentrations of BA (Figure 6(a)). In the semiquantitative analysis, ACAN in DMM + Vehicle group decreased by 47.848% compared to that in the Sham + Vehicle group. After BA treatment, ACAN in the DMM + Low-dose group was 18.342% higher than that in the DMM + Vehicle group, and ACAN in the DMM + High-dose group was higher by 41.724% than that in the DMM + Vehicle group (Figure 6(e)). For IHC staining, MMP-13 in the DMM + Vehicle group was significantly higher than that in the Sham +Vehicle group (*P* < 0.001). Although there was no significant difference in the MMP-13 content in the DMM + Low-dose group compared with that in the DMM + Vehicle group, after intervention with a high dose of BA, the content of MMP-13 decreased significantly (*P* < 0.05) (Figures 6(b) and 6(f)). We further analyzed the expression of collagen. In the DMM + Vehicle group, COL X expression was significantly increased (*P* < 0.001). A low dose of BA did not change the COL X expression in the cartilage, while the COL X levels decreased significantly after treatment with a high dose of BA (*P* < 0.01) compared to the COL X levels in the DMM + Vehicle group (Figures 6(c) and 6(g)). Additionally, COL II expression in the DMM + Vehicle group decreased by 31.614%, compared to that in the Sham + Vehicle group, and there was no significant difference in COL II expression between the DMM + Low-dose group and the DMM + Vehicle group (*P* > 0.05). The COL II expression in the DMM + High-dose group was 21.748% higher than that in the DMM + Vehicle group (Figures 6(d) and 6(h)).

### 3.6. The Elastic Modulus of DMM Mice Cartilage Increased after BA Treatment

**Figure 7:**
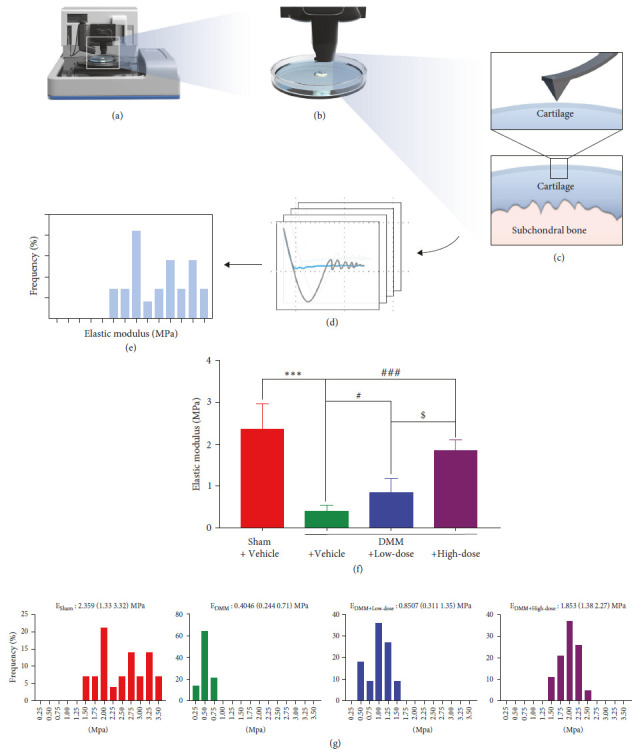
The elastic modulus of DMM mice cartilage increased after BA treatment. (a) Graphical AFM. (b) Schematic illustration of the indentation test of the cartilage in PBS solution, (c) the AFM-microindentation on the medial tibial plateau cartilage, (d) the indentation curve, (e) the *E* value distribution histograms derived at all the sites of indentation. (f) Quantification of the elastic modulus of the medial tibial plateau cartilage (*n* = 11–19). (g) The *E* value distribution histograms were derived at all the indentation sites 8 weeks after the BA treatment.  ^*∗∗∗*^*P* < 0.001 vs. Sham + Vehicle group; ^#^*P* < 0.05, and ^###^*P*<0.001 vs. DMM + Vehicle group; ^$^*P* < 0.05vs. DMM + Low-dose group.

To further verify the effects of BA on OA, we conducted a micro-indentation test on cartilage with AFM to obtain the elastic modulus (Figure 7(a)–7(e)). About 8 weeks after the treatment, the elastic modulus of the DMM + Vehicle group (*E*_DMM + Vehicle_: 0.4046 [0.244, 0.71] MPa) was significantly lower than that in the Sham + Vehicle group (*E*_Sham+Vehicle_: 2.359 [1.33, 3.32] MPa) (*P* < 0.001). After BA intervention, the elastic modulus of the DMM + Low-dose group recovered mildly (*E*_DMM + Low-dose_: 0.8507 (0.311, 1.35) MPa) (*P* < 0.05), whereas, for the DMM + High-dose group, it increased significantly (*E*_DMM + High-dose_: 1.853 (1.38, 2.27) MPa) (*P* < 0.001) (Figures 7(f) and 7(g)). We also found that the recovery of the elastic modulus was dose-dependent. The DMM + High-dose group showed significant recovery in the cartilage modulus of elasticity compared to the DMM + Low-dose group, and the difference in the elastic modulus of cartilage between the two concentration groups (low and high) was statistically significant (*P* < 0.05) (Figure 7(f)).

### 3.7. BA Treatment Attenuated Subchondral Bone Sclerosis and Relieved Abnormal Bone Metabolism in DMM-Induced OA

**Figure 8:**
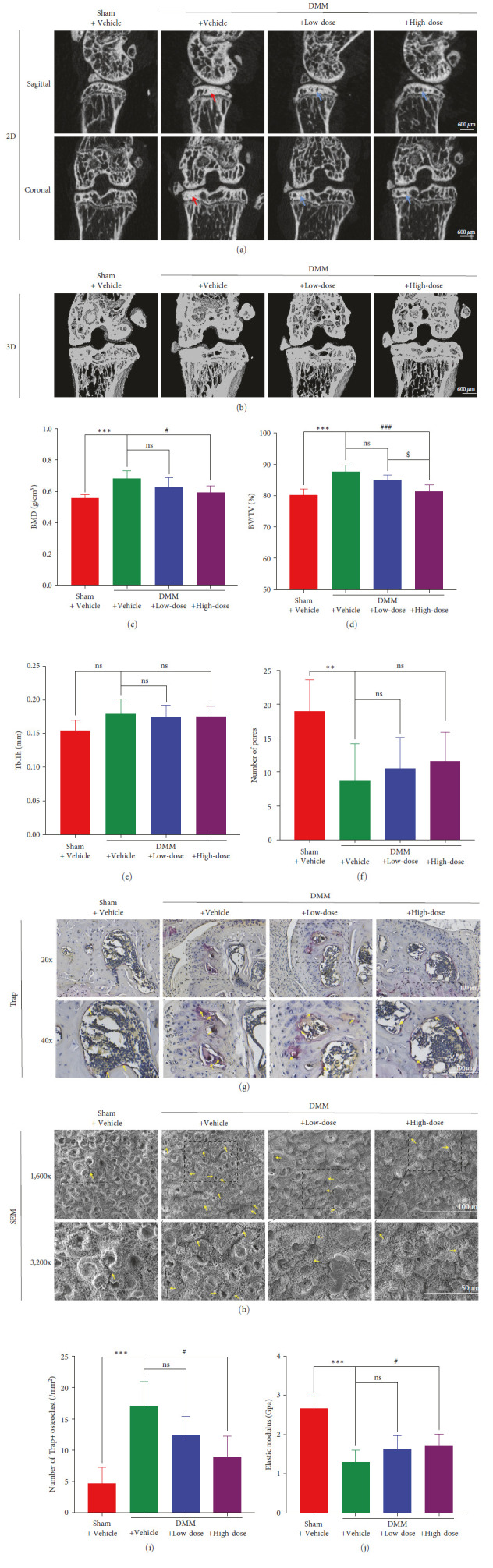
Effects of BA treatment on subchondral bone in DMM-induced OA in mice. (a) Typical 2D micro-CT micrographs of the femoral condyle and tibial plateau (sagittal and coronal views). (b) Typical 3D micro-CT micrographs of the femoral condyle and tibial plateau (coronal view). The effects of the BA treatment on subchondral bone parameters, including (c) BMD (*n* = 6–8), (d) BV/TV (*n* = 6–8), (e) Tb.Th (*n* = 6–8), and (f) number of pores (*n* = 6–8). (g) Trap staining of the subchondral bone in the right knee. (h) SEM images of the subchondral bone in the right knee. (i) Quantification of the number of Trap+ osteoclasts (*n* = 3–6). (j) Quantification of the subchondral bone modulus of elasticity (*n* = 3). Red and blue arrows, respectively, indicate osteosclerosis beneath the cartilage and subchondral articular defects, and yellow arrows indicate microfractures.  ^*∗∗*^*P* < 0.01 and  ^*∗∗∗*^*P* < 0.001 vs. Sham+ Vehicle group; ^#^*P* < 0.05 and ^###^*P* < 0.001 vs. DMM + Vehicle group; ^$^*P* < 0.05 vs. DMM + Low-dose group; ns: no significance; scale bar = 100 *µ*m.

Two-dimensional images and 3D reconstructed images of subchondral bone illustrated that DMM surgery enhanced subchondral bone sclerosis and induced the abnormal bone metabolism in murine knee joints, which were markedly attenuated by the treatment with BA (Figures 8(a) and 8(b)). According to the quantitative outcomes, BMD of the medial subchondral bone and the BV/TV ratio were significantly decreased after treatment with a high dose of BA compared to their values in the DMM + Vehicle group (*P* < 0.05 and *P* < 0.001, respectively) (Figures 8(c) and 8(d)). The BMD value and the BV/TV ratio of subchondral bone were also decreased by low-dose BA treatment, but there was no statistical significance (*P* > 0.05). After DMM surgery, the number of pores in the medial subchondral bone decreased, and BA treatment could not attenuate it significantly (Figure 8(f)). For Tb.Th, there was no significant difference among the groups (Figure 8(e)).

### 3.8. BA Decreased OCs and Microfractures of Subchondral Bone and Increased the Elastic Modulus of Subchondral Bone

For verifying the effect of BA on the subcartilage bone, we performed Trap staining on subchondral bone and observed it with AFM and SEM. Based on the results of Trap staining, the number of Trap + OCs was quantified. The DMM + Vehicle group exhibited a significantly higher number of Trap + OCs than the Sham + Vehicle group (*P* < 0.001). Although the number of Trap + OCs did not change significantly after low-dose BA intervention (*P* > 0.05), a significant reduction in the number was observed in the DMM + High-dose mice (*P* < 0.05) (Figures 8(g) and 8(i)). About 8 weeks after the DMM surgery, the subchondral bone area of ROI became rough, and the number of microfractures increased significantly. BA treatment reduced the number of microfractures in the subchondral bone area of ROI in a dose-dependent manner (Figure 8(h)). According to AFM measurements, a significant decline in the subcartilage bone modulus of elasticity was observed in the DMM + Vehicle mice (*P* < 0.001). After the intervention of low-dose BA, nonsignificant variation was found for the subcartilage bone modulus of elasticity (*P* > 0.05), while it increased significantly after high-dose BA treatment (*P* < 0.05) (Figure 8(j)).

### 3.9. BA Inhibited the Activation of NLRP3 Inflammasome in Macrophage

**Figure 9:**
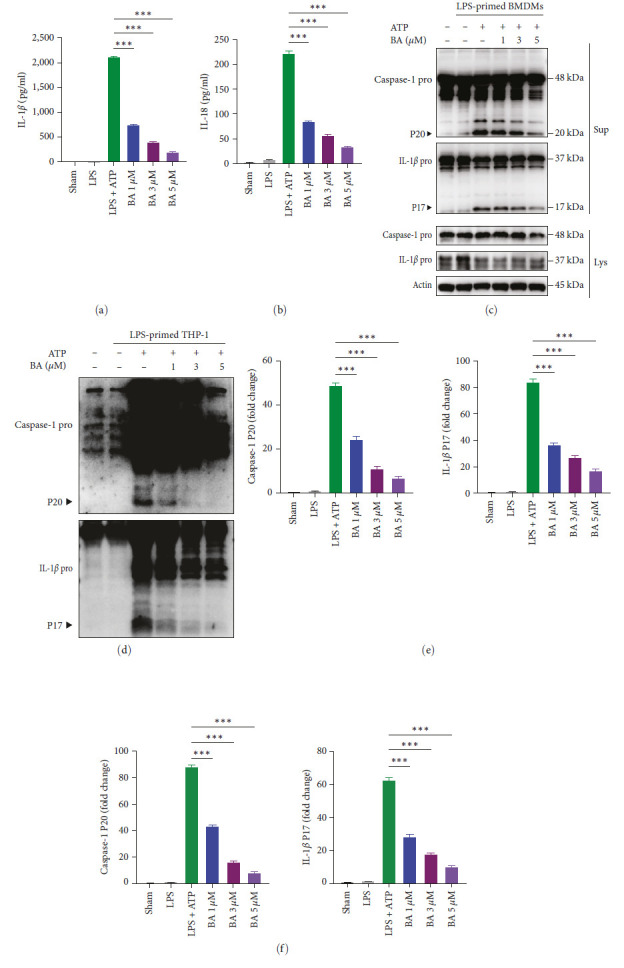
BA inhibited the activation of NLRP3 inflammsome. (a–c, f) LPS-primed BMDMs were treated with ATP or ATP + BA for 1 hr. (a) The cytokines of IL-1*β* and (b) IL-18 in supernanants were measured by ELISA. (c) Representative western bolt image of caspase-1 and IL-1*β*. (f) The quantification of active caspase-1 (P20) and active IL-1*β* (P17). (d, e) PMA-induced THP-1 macrophages were primed with LPS for 4 hr and then stimulated with ATP or ATP + BA for 1 hr. (d) Representative western bolt image of Caspase-1 and IL-1*β*. (e) The quantification of active caspase-1 (P20) and active IL-1*β* (P17).  ^*∗∗∗*^*P* < 0.001. (*n* = 3).

**Figure 10:**
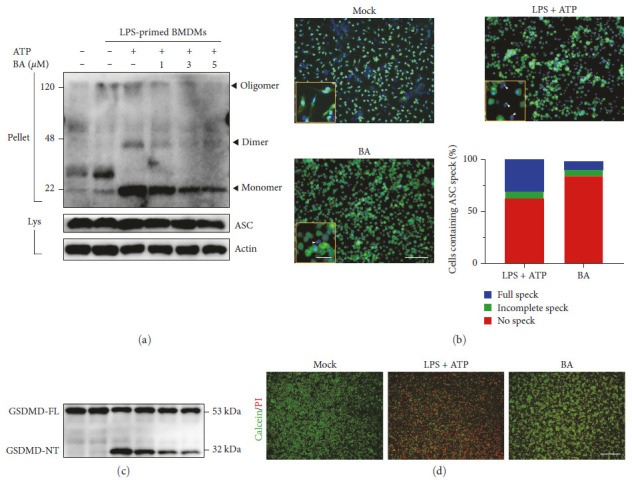
BA inhibited ASC oligomerization and speck formation. (a) Representative image of ASC oligomerization by western bolt. (b) Representative image of ASC speck formation and quantification. Scale bar 100 *µ*m (panel) 10 *µ*m (inset). (c) The analysis of GSDMD by western blot. (d) Representative image of Calcein/PI staining; scale bar = 250 *µ*m. (*n* = 3).

Given that the activation of NLRP3 inflammasome in macrophages is associated with the development of OA, we used BMDMs for in vitro study. As Figures 9(a) and 9(b) showed, BA had an inhibitory effect on IL-1*β* and IL-18 secretion in a dose-dependent manner. To confirm whether BA affected the inflammasome activation, we treated LPS-primed BMDMs with ATP (NLRP3 activator) or ATP + BA and detected the active caspase-1 (P20) and IL-1*β* (P17) in the supernanants. A similar trend of BA on inflammasome activation could be observed according to the result of western blot. When the concentration of BA comes to 5 *µ*M, the cleavage of Caspase-1(P20) and IL-1*β* could hardly be measured (Figures 9(c) and 9(f)). Furthermore, we also found that BA could dose-dependently deactivate the activation of NLRP3 inflammsome in PMA-induced THP-1 macrophages (Figures 9(d) and 9(e)). Then we investigated the underlying mechanism of BA on the inflammasome activation. The results of ASC oligomerization and speck formation revealed that BA could affect the assemble of inflammasom (Figures 10(a) and 10(b)). Additionally, pyroptosis followed by the inflammasome activation was also suppressed in the result of GSDMD (Figure 10(c)). The same result colud be observed in Calcein/PI staining with less cell deaths (Figure 10(d)). Collectively, BA alleviated OA through the inhibition of NLRP3 inflammasome activation.

## 4. Discussion

In the OA chondrocytes of humans, BA was shown to have a protective effect on IL-1*β*-triggered inflammation [21]. Similarly, Wang et al. [34] found that in a rat model of sodium monoiodoacetate (MIA)-triggered OA, the BA derivatives fuzed with C-17-amino-substituted pyrazole had a therapeutic effect which reduced the joint pain, mitigated cartilage destruction, and osseous changes. We found that BA treatment improved the condition of the DMM-induced OA mouse model by inhibiting matrix degradation, preventing subcartilage bone remodeling, and mitigating synovitis.

In the progression of OA, in cartilage, the changes in chondrocytes trigger matrix degradation, resulting in the destruction of the cartilage [9, 10]. For the subchondral bone, the balance of osteoblast and OC was broken, resulting in the subchondral bone remodeling [35–37]. In the synovial membrane, there was an increase in the infiltration of inflammatory cells [38, 39]. We found drastically reduced ACAN, COL II levels, as well as sulfated GAG chains of proteoglycans in the cartilage of DMM-induced OA mice. However, the expression of MMP-13 and COL X was significantly upregulated, which was consistent with the results of previous studies [40–42]. BA treatment could dose-dependently reverse these changes. COMP is a crucial ECM protein and a key biomarker of ECM hydrolysis [43, 44]. Sharif et al. [45] showed that the level of serum COMP is correlated with the level of joint injury in knee OA; the more severe the joint damage, the higher the COMP content. In this study, although the serum COMP level of mice increased after DMM-induced OA surgery, it decreased after BA treatment. This also proved that BA intervention could alleviate the degeneration of the articular cartilage. Additionally, we used AFM to quantify the elastic modulus of cartilage and found that DMM surgery reduced the elastic modulus of cartilage, which was alleviated by the BA treatment. We also found a significant increase in BMD, the BV/TV ratio, and Trap + OC counts and microfractures in the subcartilage bone of the DMM mice. The studies by Hou et al. [3] and Muschter et al. [46] also supported these results. BA treatment suppressed these changes in a dose-dependent manner, suggesting that BA has a therapeutic effect on OA-triggered subchondral bone reconstruction. Our results also indicated that BA intervention alleviated synovial inflammation caused by the DMM surgery.

Several studies have shown the association of NLRP3 inflammasome initiation with the progression of OA. For example, DMM-induced OA was shown to activate the expression of NLRP3 inflammasome [13, 47, 48]. Specifically, NLRP3 inflammasome activates caspase-1, causing maturation of pro-inflammatory IL-1*β* and IL-18 cytokines into their respective active forms, leading to further inflammatory responses [49, 50]. Moreover, IL-1*β* can induce the cartilage destruction by suppressing ACAN and collagen synthesis in chondrocytes and upregulating ADAMTS-4 and MMP-13 [17, 51–54]. From our in vitro experiments, we found that BA treatment could dose-dependently deactivate the activation of the NLRP3 inflammasome, which reduces the activation of caspase-1. After the reduction of caspase-1, the production of GSDMD-NT was also reduced, leading to a decrease in the maturation and secretion of IL-1*β*. This beneficial effect was also observed in the serum by performing ELISA for IL-1*β*. Additionally, several studies have shown that the activation of NLRP3 inflammasome can promote OC differentiation, which was consistent with our results [55, 56]. Previous study has shown that BA can inhibit OC differentiation in vitro mainly through MAPK and NFATc1 signaling pathways [57]. Tumor necrosis factor-alpha (TNF-*α*) can sequentially activate NF-*κ*B, c-Fos, and NFATc1 to induce the OC formation [58]. NFATc1 and c-Fos are key effectors in MAPK signaling [59]. While IL-1 alone can not induce the OC formation, and IL-1*β* mediates TNF-induced osteoclastogenesis independent of NF-*κ*B [60, 61]. Therefore, we speculate that there is no direct relationship between MAPK and NFATc1 signaling and IL-1*β*. Collectively, this study has showed that BA could reduce the maturation and secretion of IL-1*β* by limiting NLRP3 inflammasome initiation as well as pyroptosis.

Although our study explains the effect of BA on OA to some extent, there are still many deficiencies. First, we could not obtain enough samples to perform cytokines, such as IL-1*β* and TNF-*α*, due to the lack of synovial fluid in the joint of mouse. Second, previous study has demonstrated that BA can inhibit TNF-*α*-stimulated inflammatory effects to alleviate the symptoms of OA [62]. It is with regret that we did not have enough time to explore the mechanisms. So, further research is required to extend our findings and improve the treatment of OA.

## 5. Conclusions

**Figure 11:**
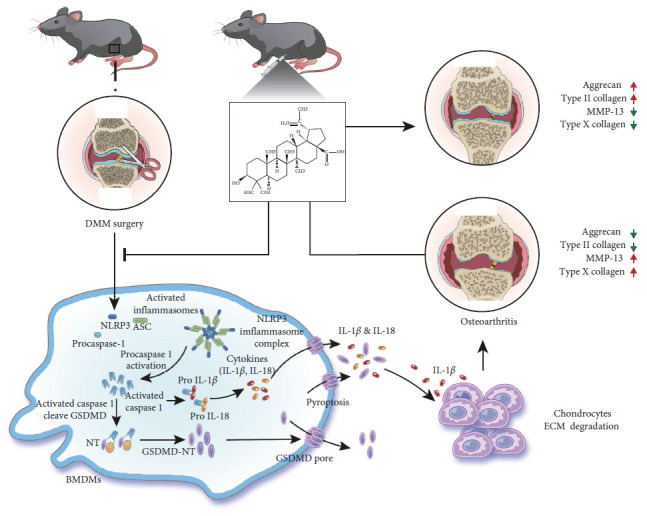
BA attenuates OA via limiting NLRP3 inflammasome activation to decrease IL-1*β* maturation and secretion.

In summary, this study suggested that BA can protect against DMM-induced OA, the mechanisms may be related to BA can limit NLRP3 inflammasome activation to decrease the IL-1*β* maturation and secretion (Figure 11). The results highlighted that BA has a promising pharmacological value in postponing the progression of OA, which is worthy of further investigation.

## Data Availability

The datasets used and analyzed during the current study are available from the corresponding author on reasonable request.

## Abbreviations

OA: Osteoarthritis
IL-1*β*: Interleukin-1*β*
BA: Betulinic acid
SEM: Scanning electron microscopy
IHC: Immunohistological
IF: Immunofluorescence
AFM: Atomic force microscopy
BMDMs: Bone marrow-derived macrophages
ECM: Extracellular matrix
ACAN: Aggrecan
COL II: Collagen II
COL X: Collagen X
MMP-13: Matrix metalloproteinase-13
GSDMD: Gasdermin D
DMM: Destabilization of the medial meniscus
BOS: Base of support
COMP: Eartilage oligomeric matrix protein
ROI: Regions of interest
BV/TV: Volumetric ratio of bone to tissues
BMD: Bone mineral density
Tb.Th: Thickness of trabecula
EDTA: Ethylenediaminetetraacetic acid
H&E: Hematoxylin–eosin
Trap: Tartrate-resistant acid phosphatase
HC/CC: Ratio of hyaline cartilage to calcified cartilage
OARSI: The Osteoarthritis Research Society International
OC: Osteoclast
TNF-*α*: Tumor necrosis factor-alpha.
